# Principal Leadership Role in Response to the Pandemic Impact on School Process

**DOI:** 10.3389/fpsyg.2022.943442

**Published:** 2022-07-11

**Authors:** Philip Saagyum Dare, Atif Saleem

**Affiliations:** ^1^Faculty of Education, Monash University, Melbourne, VIC, Australia; ^2^College of Teacher Education, Zhejiang Normal University, Jinhua, China

**Keywords:** school leadership, principal, emotional intelligence, managerial factors, organization process, COVID-19

## Introduction

A school constitutes a complex bureaucratic managerial organization with decentralized services set up by relevant societal laws that draw and exert influence from both regional and global communities. Like most societal organizations, schools are led by school principals or leaders. The behavior of a school principal is crucial in determining the general school performance (Constantia et al., 2021) and its members, students, teaching, and non-teaching staff alike. In education, the principals plan school life, maintain legislation and education ministry circulars, provide official directives, and implement teacher-related activities (Edo et al., 2019). Principals are responsible for developing and implementing curriculum growth and facilitating staff and students' operations by motivating and guiding them along with the requirements of school goals (Constantia et al., 2021). Therefore, one may argue that school leadership is sufficiently variable and replicates a combination of characteristics. On the other hand, school principals are expected to meet the expectations of parents, teachers, and students by being creative and imaginative, solving problems, and engaging with families and indigenous people (Constantia et al., 2021).

**Figure 1:**
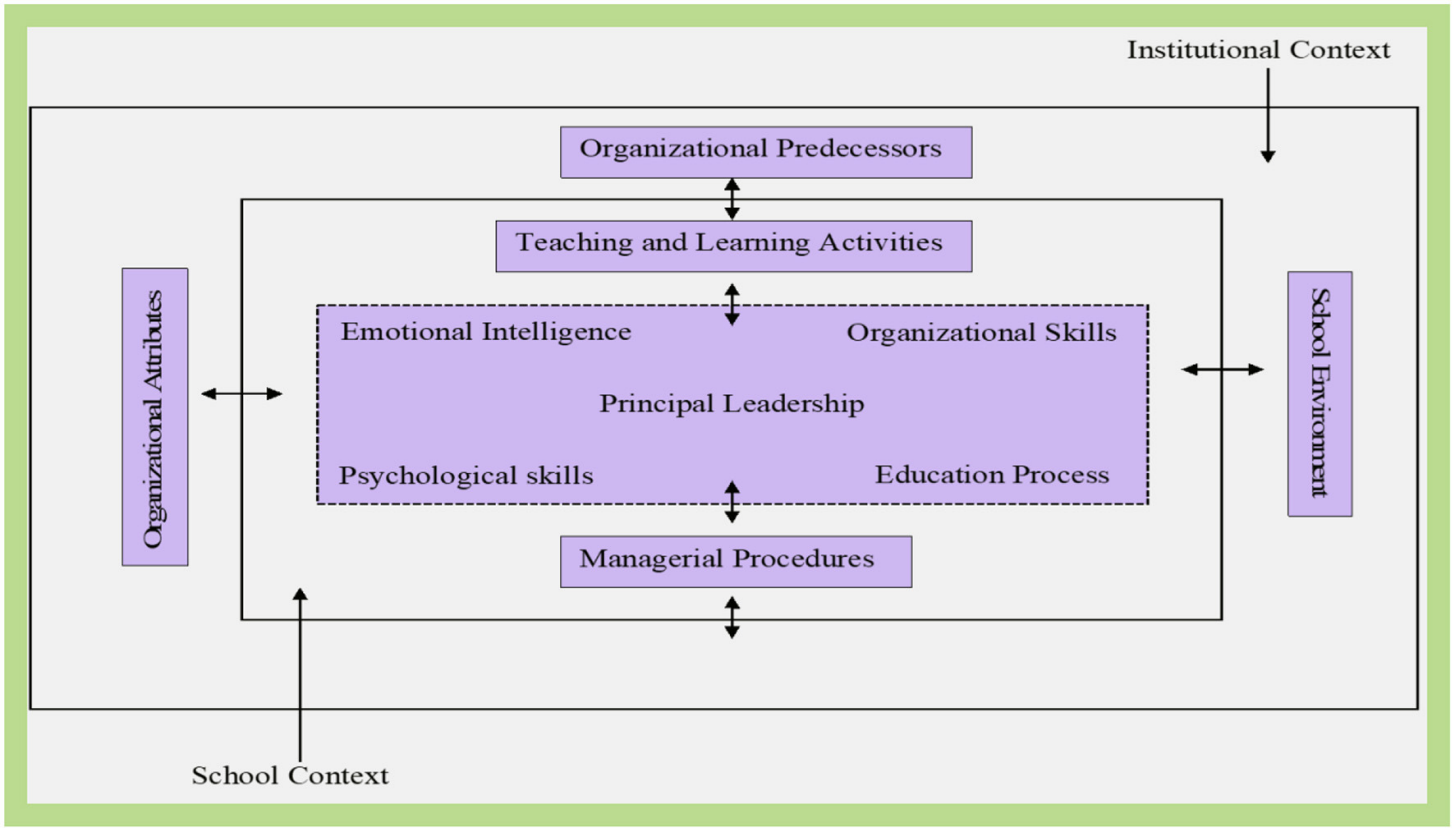
Principal leadership responsiveness.

School principals' roles in improving the quality of education for all include the following: (a) enhancing school improvement by improving learning quality among students; (b) reinforcing and motivating students, teaching and non-teaching staff to participate in the process of improvement; (c) prioritizing and promoting the notion that everyone within the school community should work toward the change process; and finally (d) making provisions for an enviable learning environment. Therefore, the principal is not merely a school manager but the major variable determining change within schools as organizations (Figure 1). To be successful, school leaders should be well-equipped with features as they spearhead the respective school institutions and their employees (Jennings et al., 2019). The principal must be intimately knowledgeable of these characteristics to integrate them into the workplace skillfully (Leithwood et al., 2008). In addition, school principals are equally accountable for school projects' implementation and innovations. However, the school systems were barely equipped for the disruptive effects of the COVID-19 virus outbreak.

## COVID-19 Epidemics

During such a lengthy crisis, it is likely that long-term demands will exceed the personal strengths and employment resources (Bakker and Demerouti, 2017) of school leaders, thus making them vulnerable to their leadership roles; home-school navigation; weariness; and high burnout risks of both administrative and managerial roles (Kniffin et al., 2021). The COVID-19 pandemic is unprecedented, and most school principals do not have expertise in dealing with such protracted-complex crises (Varela and Fedynich, 2020). In March 2020, when WHO declared the SARS-CoV-2 outbreak a pandemic, normal school routines, operations, and processes were dramatically interrupted; schools were forced to implement improvised strategies to manage children and staff's safety and, at the same time, slow down the transmission of the virus (Bailey and Breslin, 2021).

In this regard, the present opinion article presents an eye view of commentary on principals' leadership roles in response to the pandemic impact on the school process. Specifically, we reviewed the impact of the pandemic on the school systems of change and the school principals' leadership roles in the transitioning process. As nations shut down schools, educational activities were shifted swiftly to virtual and digital platforms (Taglietti et al., 2021), with corresponding challenges such as inadequate and available virtually-related materials, preparations, and resources. Thus, school principals had to devise different ways of handling the situation within their leadership roles.

## School Leadership During COVID-19 Pandemic

In the past year, the COVID-19 plague significantly influenced our daily lives (Azorín and Fullan, 2022). In order to control the spread of infectious diseases worldwide, tight limitations were implemented everywhere (Harris and Jones, 2020), impacting many parts of human existence, particularly school systems, which are now required to reform their instructional responsibilities and methods (Zhao, 2020). School leadership refers to a process that determines the educational climate. Therefore, it must devise strategies to respond to socioeconomic and cultural circumstances (Alhouti, 2020) and the pandemic's consequences. In such circumstances, school principals had to operate virtually quickly, efficiently, precisely, and appropriately (Harris and Jones, 2020; Netolicky, 2020) to maximize the educational goals affected by the plague. Unfortunately, these school leaders are met with numerous unanticipated situations that require immediate and remote remedy. Harris and Jones (2020) claimed that the unavailability of human interactions in regular forms, in-person interactions between and among school principals, families, teachers, and students, including the larger school environment, constitutes some of the principals' most significant challenges during the plague.

It is impossible to disregard the core relevance of school principals, their leader's compelling vision, and professional growth (Leithwood et al., 2008; Harris and Jones, 2020) during this COVID-19 pandemic. Studies that investigated school principals' leadership responses to the pandemic effects on these educational processes suggested that school leaders used emotionally-related leadership behaviors and intelligence. School leaders must examine and address the emotional needs of teachers and students. Constantia et al. (2021) suggest that fear predominantly determines the emotionality of students during the transition of school systems. The authors also noted that students also experienced anxiety and perplexity due to fear experiences. Therefore, principals should be trained in times of crisis difficulties, like the COVID-19 pandemic, including issues relating to remote instructional technology.

Moreover, Ravitch (2020) indicated that pandemics had worsened pupils' emotions of exclusion as school principals' roles grew untenable with exuberant demands to comply with departmental education circulars, safety procedures, and rules. Sadly, school leaders must handle all of these with no prior experience, training, or induction from educational leaders.

## School Principals' Emotionally-Related Leadership Behaviors and Responses to the Pandemic Effects

The principals' efforts to deliver blended learning may be bolstered if emotional intelligence is employed in the viewpoint of studies. Bar-On (2000) described emotional intelligence as a combination of emotional and social competencies, adaptive skills, and personal characteristics to lead others. Based on the important role emotional intelligence plays, especially in times of crisis, Aldiabat (2019) described psychological skill as a crucial component of a strong leader because it involves self-awareness, self-control, social intelligence, and management of interpersonal interactions.

In commending this proposition, Doe et al. (2015) suggest that emotional intelligence is a critical attribute of a competent leader, i.e., the capacity to recognize and self-regulate emotions and those of others, including applying empathetic compassion as a managerial tool (Figure 1). With the skills of emotional intelligence, a school principal can positively influence the culture of the entire school population and community. Moore (2009) asserts that when a school leader motivates both instructors and pupils by tapping into their emotional maturity, they can achieve educational objectives more effectively. As a result of the school closure, the principal's emotional intelligence was no longer utilized within the school management system. There were no in-person contacts between school leaders and staff; rather, interpersonal interaction depends on technology. Unfortunately, both students and teachers, including the school authorities, may have fewer technical capabilities to manage the pandemic-driven increase in remote education. However, Constantia et al. (2021) contested that school principals' emotionally-related behaviors and or leadership attribute, when present pose significant effects on school leadership and management in times of crisis.

## Conclusion

The scoping review of the study showed that during the shutdown and consequently the remote learning, the process of learning and the responsibilities of the school principals had changed. This is consistent with the findings of Zhao (2020), who demonstrated that the COVID-19 pandemic has significantly impacted the teaching-learning activities and, consequently, the principals' roles across the board. Specifically, this guide leads us to the conclusion that one of the issues that develop due to pandemic lockdown and remote teaching includes an increase in regulatory work, which perpetuates the already depressed school climate and badly impacts the organizational skills and managerial skills activities of principals.

The tough nature of resolving technical issues that followed the teaching and learning process constituted another obstacle that school systems confronted. This arguably occurred because school leaders and teachers were unfamiliar with the electronic medium utilized for remote education. Moreover, the social distance exists inside the school environment. Students had trouble concentrating throughout the online class since there was insufficient contact between the principals and the respective teacher association. This is consistent with the findings of Harris and Jones (2020). They concluded that no interaction existed between teachers and school principals as teaching instructions and general school routines were virtually disseminated without dialogue.

School principals and teaching and non-teaching staff can manage the described challenges in various ways. First, school principals should encourage collaboration among teacher associations and emphasize the significance of motivation by urging teachers to create dynamic lessons that enhance virtual interactions among students. These kinds of situations can improve the emotional intelligence and skills of school principals, teachers, other staff, and students (Quisenberry, 2018; Ma et al., 2019). The school leaders hereby become bureaucrats who become lost in directives, circulars, and health procedures meant to promote the effective regulation of the school during the pandemic. As school principals act as leaders with a shared vision among and with the teacher's organization and an administrative delegate from the education ministry, the principal's involvement in managing the pandemic situation alongside the school is vital.

School leaders can organize seminars for teachers, families, and learners on digital literacy using distance learning platforms and teenage psychology. As the study's participants suggested, boosting the principal's emotional intelligence can be a very effective “tool” for resolving the crisis. Considering the relevance of the findings identified from various studies, we acknowledge some limitations. Therefore, we suggest that future studies should employ a mixed method research technique to strengthen the validity of the conclusions drawn on the relevance of school principals' emotional leadership behaviors to the educational process in times of pandemic.

In recent decades, the global school community has argued at length about the role of school principals' in ensuring the institution's efficient running. Studies indicated, *via* a historical examination of school leadership, that the role of the school leader is continuously molded and adjusted to the present socio-cultural and economic conditions, including times of crisis. As a result of the COVID-19 pandemic, the global school leadership has been restructured. Considering the impacts of the pandemic on the educational process, this paper examines the role of school principals (Figure 1). Using emotional intelligence and leadership behaviors to mitigate the pandemic was an effective way for principals to deal with the situation.

## Author Contributions

All authors listed have made a substantial, direct, and intellectual contribution to the work and approved it for publication.

## Conflict of Interest

The authors declare that the research was conducted in the absence of any commercial or financial relationships that could be construed as a potential conflict of interest.

## Publisher's Note

All claims expressed in this article are solely those of the authors and do not necessarily represent those of their affiliated organizations, or those of the publisher, the editors and the reviewers. Any product that may be evaluated in this article, or claim that may be made by its manufacturer, is not guaranteed or endorsed by the publisher.
